# Contributions of Adaptive Plant Architecture to Transgressive Salinity Tolerance in Recombinant Inbred Lines of Rice: Molecular Mechanisms Based on Transcriptional Networks

**DOI:** 10.3389/fgene.2020.594569

**Published:** 2020-10-23

**Authors:** Isaiah Catalino M. Pabuayon, Ai Kitazumi, Glenn B. Gregorio, Rakesh Kumar Singh, Benildo G. de los Reyes

**Affiliations:** ^1^Department of Plant and Soil Science, Texas Tech University, Lubbock, TX, United States; ^2^International Rice Research Institute, Los Baños, Philippines

**Keywords:** genetic novelty, transgressive segregation, salinity, plant architecture, growth habit, network rewiring

## Abstract

Genetic novelties are important nucleators of adaptive speciation. Transgressive segregation is a major mechanism that creates genetic novelties with morphological and developmental attributes that confer adaptive advantages in certain environments. This study examined the morpho-developmental and physiological profiles of recombinant inbred lines (RILs) from the salt-sensitive IR29 and salt-tolerant Pokkali rice, representing the total range of salt tolerance including the outliers at both ends of the spectrum. Morpho-developmental and physiological profiles were integrated with a hypothesis-driven interrogation of mRNA and miRNA transcriptomes to uncover the critical genetic networks that have been rewired for novel adaptive architecture. The transgressive super-tolerant FL510 had a characteristic small tiller angle and wider, more erect, sturdier, and darker green leaves. This unique morphology resulted in lower transpiration rate, which also conferred a special ability to retain water more efficiently for osmotic avoidance. The unique ability for water retention conferred by such adaptive morphology appeared to enhance the efficacy of defenses mediated by Na^+^ exclusion mechanism (*SalTol*-effects) inherited from Pokkali. The super-tolerant FL510 and super-sensitive FL499 had the smallest proportions of differentially expressed genes with little overlaps. Genes that were steadily upregulated in FL510 comprised a putative cytokinin-regulated genetic network that appeared to maintain robust growth under salt stress through well-orchestrated cell wall biogenesis and cell expansion, likely through major regulatory (*OsRR23, OsHK5*) and biosynthetic (*OsIPT9*) genes in the cytokinin signaling pathway. Meanwhile, a constitutively expressed cluster in FL510 prominently featured two transcription factors (*OsIBH1, TAC3*) that control tiller angle and growth habit through the brassinosteroid signaling pathway. Both the putative cytokinin-mediated and brassinosteroid-mediated clusters appeared to function as highly coordinated network synergies in FL510. In contrast, both networks appeared to be sub-optimal and inferior in the other RILs and parents as they were disjointed and highly fragmented. Transgressively expressed miRNAs (*miR169, miR397, miR827*) were also identified as prominent signatures of FL510, with functional implications to mechanisms that support robust growth, homeostasis, and osmotic stress avoidance. Results of this study demonstrate how genetic recombination creates novel morphology that complements inducible defenses hence transgressive adaptive phenotypes.

## Introduction

Adaptation to the dynamic nature of abiotic and biotic factors in the environment is crucial for plant survival and reproduction (Anderson et al., 2011). Success in an ecological niche is optimized by environmental pressures that interface with genetic and epigenetic potentials across a founder population by virtue of genotype-by-environment interaction (GxE). The GxE dynamics translate into adaptive growth, developmental and reproductive habits through intricately programmed networks of gene expression (Van Wallendael et al., 2019). Recent studies supported the role of natural hybridization between genetically distant parents as important mechanism for the creation of rare developmental, morphological, and/or adaptive traits that are outliers from the majority of the population, i.e., genetic novelties. These novelties have been proposed to set foundations for new phylogenetic lineages that adapt to new ecological niches (Wagner and Lynch, 2010).

Engineering the new generation of agricultural crops with adaptive capacity to emerging ecological dynamics amidst the marginalization created by climate change, natural resource deterioration, and environmental degradation has become the core of plant breeding in the 21st century. Similar to ecological and evolutionary dynamics, key to this goal are novel phenotypes that are above and beyond what has already been achieved by earlier breeding paradigms (de Los Reyes, 2019). For instance, the Green Revolution in the 1960’s was hallmarked by the creation of novel plant architecture that was optimal for managed environments with ideal water and nutrient inputs. In rice, pivotal to the success of the Green Revolution was the novel semi-dwarf architecture conferred by the *sd1* mutation, with lower than normal levels of bioactive gibberellic acid (GA) that prevented extensive stem elongation (De Datta et al., 1968; Chandler, 1992; Monna et al., 2002; Sasaki et al., 2002; Hedden, 2003; Peng et al., 2010). Semi-dwarf cultivars exhibited a special adaptive trait that avoided lodging, which is a negative offshoot of enhanced yield created by efficient mobilization of resources from vegetative source to reproductive sinks. Increased seed yield would otherwise have negative pre-harvest impacts in the native landraces with tall and lanky stature, because of inability to support the heavy weight of the panicles created by efficient source-sink partitioning. Therefore, ideotype breeding created an adaptive plant architecture for water-rich and nutrient-rich environments (Vergara, 1988; Khush, 1995a).

While the Green Revolution enabled dramatic improvements in yield, it also led to dramatic increase in water and nutrient requirements to support the full genetic potentials of modern cultivars. The semi-dwarf, high-yielding cultivars had a narrow ecological niche beyond well-managed agricultural ecosystems. For instance, *sd1* is linked to an inferior allele of the drought tolerance QTL *qDTY_1_._1_* that is important for maintaining yield in upland landraces, which normally thrive with limited water (Vikram et al., 2016). While the successful modification of rice ideotype through *sd1* created a large net gain in grain yield under well-managed environments, the technology also led to unanticipated trade-offs because of the loss of other attributes that would allow a wider ecological adaptive capacity. There is a renewed vision to recover the lost attributes to create the 21st century breeds of crops with minimal yield penalty under marginalized environments (Khush, 2001; Khush, 2005).

In response to the paradigm switch triggered by the burgeoning ecological threats to agriculture, in the 1990’s, the International Rice Research Institute (IRRI) proposed to remodel the developmental and morphological architecture of the rice plant, to create the ‘*new plant-type*’ (NPT) for enhanced performance to more limiting ecological settings. The aim was to create a new ideotype with fewer but fully productive tillers, sturdy stems, dark green, thick, and erect leaves, and vigorous roots, toward even greater increases in harvest index (Peng et al., 1994; Khush, 1995b). The Green Revolution varieties typically produce 30–40 tillers, of which only half produce panicles even under water-rich and nutrient-rich environments. Such ideotype creates issues with nutrient allocation, with vegetative tillers creating a *‘sink’* for nutrients instead of *‘source’* for seed development. Thus, the NPT combined the features that prevented pre-harvest losses through lodging, fine-tuned biomass production for channeling photosynthate toward grain development, and suppressed unproductive vegetative growth, giving as much as 1.5 tons ha^–1^ improvement in yield (Khush, 2013). The NPT represents another example of the importance of adaptive domestication by breeding to address the burgeoning environmental threats to modern agriculture.

The phenomenon of transgressive segregation, which is observed in both natural and artificial populations created by plant breeding, is characterized by the occurrence of minority phenotypic outliers relative to parental range across a segregating or recombinant population derived from genetically divergent parents. In addition to the classic explanations attributing complementation and epistatic interactions as major mechanisms behind transgressive traits, the possible roles of coupling and uncoupling effects and genetic network rewiring have also been recently proposed (Vega and Frey, 1980; Rieseberg et al., 1999; Dittrich-Reed and Fitzpatrick, 2013; de Los Reyes, 2019). Combined with the paradigms of genomic biology, the potential of transgressive individuals for enhanced yield of crops have been established, but its true potential for adaptive traits is yet to be determined (DeVicente and Tanksley, 1993).

A modern view on phenotypic variance for complex traits was recently presented by the *Omnigenic Theory* through the synergistic interaction between the relatively fewer major-effect ‘*core genes*’ and the more numerous but minute-effect ‘*peripheral genes*’ scattered throughout the genome (Boyle et al., 2017). A further extension of the *Omnigenic Theory* may also accommodate the potential roles of not just the protein-coding but also the non-protein-coding regions (ncRNAs) of the genome as both core and peripheral components (Carrington and Ambros, 2003; Mallory and Vaucheret, 2006; Khraiwesh et al., 2012). This study represents a holistic re-examination of the phenomenon of transgressive segregation for an adaptive trait (salt tolerance) in rice by integrating the potential contributions of both transcriptional and post-transcriptional regulatory mechanisms. Guided by the visions of the *Omnigenic Theory*, the aim of this study was to illustrate the molecular synergies in context of the genetic network paradigm behind the outlier stress-adaptive phenotypes across a recombinant inbred (RIL) population derived from the improved high-yielding cultivar IR29 (salt-sensitive) and an Indian landrace Pokkali (salt-tolerant; Pabuayon et al., 2020).

Results showed that salt stress defense capacity encoded by the *SalTol* QTL was not the main driver behind transgressive super-tolerance or super-sensitivity (Pabuayon et al., 2020). Rather, transgressive salt-tolerance can be explained to larger extents by rewired genetic networks that involved regulatory transcription factors and miRNAs that determine plant architecture, growth habit, and stress avoidance. This study provides yet another example that the optimization of adaptive potential requires the modification not only of the capacity for defense and repair but more importantly, the reconfiguration of developmental attributes to allow growth adjustment and stress avoidance, similar to the new ideotypes created by rice breeding during the Green Revolution and post-Green Revolution era.

## Materials and Methods

### Plant Growth Conditions

The comparative panels of genotypes used in this study were selected from earlier studies that identified with transgressive segregants at EC = 12 dS m^–1^ during V4–V12 stage of vegetative growth (Pabuayon et al., 2020). Four recombinant inbred lines (RILs) derived from IR29 (*Xian/indica*; salt-sensitive) × Pokkali (*Aus*; salt-tolerant) representing different levels of salinity tolerance were used for morpho-developmental profiling and RNA-Seq experiments. These included FL510 (super-tolerant), FL499 (super-sensitive), FL478 (tolerant), and FL454 (sensitive). Plants were grown in Lubbock, Texas under standard greenhouse conditions at 30–35°C day, 24–26°C night; 20 to 30% RH; 12-hour photoperiod with an average light intensity of 500 μmol m^–2^s^–1^ (photosynthetic photon flux density).

Seeds were germinated at 30°C in petri dishes lined with wet filter paper for 2 days and grown in seedling trays with standard peat moss potting mix for 14 days. Individual plants were transplanted to 2.27-liter (0.6-gallon) hydroponic buckets with 1 g L^–1^ Peter’s Professional 20-20-20 General Fertilizer at pH 5.8 supplemented with 0.4 g L^–1^ FeSO_4_⋅7H_2_O. The plants were grown until maximum tillering stage (V10–V12; Counce et al., 2000), and salinity stress was introduced by increasing the electrical conductivity (EC) of the hydroponic solution to EC = 12 ds m^–1^ (∼120 mM) with NaCl. Tissue samples (shoots) for RNA extraction were collected prior to stress (control; 0 h) and 24, 48, 72, and 144 h after stress.

Separate sets of plants were used for morpho-developmental and physiological profiling. For these measurements, salinity stress was not imposed. Plants were grown to their maximum potential to enable precise description of morphology and growth habit without stress limitations, thus allowing the examination of the differences in inherent morpho-developmental potentials in relation to their salinity tolerance capacities. Individual seedlings were transplanted into buckets with Turface MVP^®^ and submerged in the hydroponics solution. Plants were grown to the same maximum tillering stage as in the transcriptome experiment described above (V10–V12).

### Morpho-Developmental and Physiological Profiling and Statistical Analysis

The overall vegetative morphology and growth habit of each genotype in the comparative panel were characterized. Width of leaf blades was measured at the widest point (middle area) using a digital caliper. Three leaves per plant were measured at random, and six plants were used for this analysis (*n* = 18). Tiller angle and plant height were measured via image analysis using Fiji software (Schindelin et al., 2012). The furthest tiller was used as a reference point and its angle from the base of the plant from the vertical axis perpendicular to the ground was measured. Plant height was measured as the length from the visible base to its highest point (*n* = 6). Shoot and root biomass was measured from three individual plants. Samples were oven dried at 70°C for at least 5 days and weighed. Tiller number was counted as the number of individual tillers stemming from the base of the plant shoot (*n* = 6). Stomatal conductance was measured in terms of flux (mmol/m^2^s, *n* = 5) using the SC-1 Leaf Porometer (Meter Group, Inc., Pullman, WA, United States).

Statistical analysis was conducted on R 4.0.1 (R Core Team, 2020). Analysis of Variance (ANOVA) and Tukey’s HSD *post hoc* tests were conducted using the ‘agricolae’ package with significance tests set at *P* < 0.05 (De Mendiburu and De Mendiburu, 2019). Data was visualized using the ‘ggplot2’ package (Wickham and Chang, 2008). The clustering dendrogram and heatmap was made with the package ‘gplots’ with default parameters (Warnes et al., 2016).

### RNA-Seq and Transcriptional Network Modeling

The temporal transcriptome experiments were designed to capture both the immediate (24, 48, 72 h) and long-term (144 h) responses to salt stress relative to control (0 h) to enable dissection of different patterns of co-expression through a temporal and inter-genotypic transcriptome matrix. Parallel libraries were constructed to capture both the mRNA and sRNA/miRNA reads by RNA-Seq. Total RNA was extracted from frozen leaf tissues using miRVana^TM^ miRNA Isolation Kit (Invitrogen, Carlsbad, CA, United States) and used to construct time-course (0, 24, 48, 72, 144 h) RNA-Seq libraries. The sRNA/miRNA libraries were also made from the same RNA samples using Bioo Scientific NextFlex^TM^ Small RNA-seq Kit V3 (Bioo Scientific Corporation, Austin, TX, United States). Strand-specific 150-bp paired-end (mRNA) and single-read (sRNA/miRNA) RNA-Seq libraries were sequenced on Illumina HiSeq3000 with two replicates (Oklahoma Medical Research Foundation, OK, United States). Sequence output from mRNA-Seq and sRNA-Seq libraries (PRJNA378253) were preprocessed with Cutadapt (v2.10) and mapped against the Nipponbare genome (IRGSP-1.0) and miRBase (*Oryza sativa* mature miRNA, v22.1) using HISAT2 (v2.1.0) and blat (v36), respectively (Kent, 2002; Fahlgren et al., 2010; Martin, 2011; Sakai et al., 2013; Kim et al., 2015; Stark et al., 2019). Transcript read counts were normalized by trimmed mean M-values (TMM) by Subread (v1.5.2) and differential expression was examined by edgeR (v3.24.3) using a false detection rate of 0.05 (McCarthy et al., 2012; Liao et al., 2013). Expression values were analyzed and shown as the log2 fold-change (log2-FC) relative to control for each genotype, allowing the comparative analysis of expression patterns among the different genotypes.

Assignment of genes to relevant metabolic pathways and gene ontologies were done through the Kyoto Encyclopedia of Genes and Genomes (Kanehisa and Goto, 2000; Moriya et al., 2007). Enrichment of GO terms was calculated with R package ‘goseq’ and a cutoff of *P* < *0.05* was set for identifying significant ontologies (Young et al., 2010). Visualization of data and computation of relevant Pearson’s correlation coefficient (PCC) matrices were performed with R 4.0.1 (R Core Team, 2020). PCC was used to determine the strength and direction of the relationship between genes. This information was then used to create gene networks. As there was no specific central gene selected, correlation coefficients were used as a threshold for filtering connections (edges) between genes (nodes). This allowed the discernment between co-expression and reverse co-expression of genes and to demonstrate the cohesion or fragmentation of a gene network in a genotype. Networks were constructed and visualized using Cytoscape (Smoot et al., 2010).

Upset graphs were created using the package ‘UpsetR’ (Conway et al., 2017) and were used to show the distribution of genes in different classes. Heatmaps were created using the package ‘gplots’ (Warnes et al., 2016). Alluvial graphs were created using the ‘alluvial’ package (Bojanowski and Edwards, 2018) to show the similarity of gene expression patterns between genotypes.

## Results

### Morphological and Developmental Novelties of Transgressive Segregants From IR29 × Pokkali

Transgresssive segregation for salt tolerance was uncovered in a recombinant inbred population (F_8_-RIL) of rice derived from the improved high-yielding *Xian/Indica* cultivar IR29 (salt-sensitive) and the Indian *Aus* landrace Pokkali (salt-tolerant), based on integrative molecular, biochemical, macro-physiological, and real-time growth profiling approach to phenotyping (Pabuayon et al., 2020). Pokkali is a known donor of seedling-stage (V2–V4) salt-tolerance by the Na^+^ exclusion mechanism encoded by the *SalTol* QTL (Thomson et al., 2010). Systems-level characterization of each individual across the population identified two representative F_8_-RILs as clear outliers relative to parental range of growth potential under salt stress. FL510 represents a positive transgressive (super-tolerant) progeny and FL499 represents a negative transgressive (super-sensitive) progeny. Two other sibling RILs, FL478, and FL454, represented the salt-tolerance similar to the donor parent Pokkali and salt-sensitivity similar to the recipient parent IR29, respectively. Along with the parents, these four sibling RILs (salt-tolerant FL478, salt-sensitive FL454, super-tolerant FL510, and super-sensitive FL499) comprised the minimal comparative panel for in-depth characterization.

**FIGURE 1:**
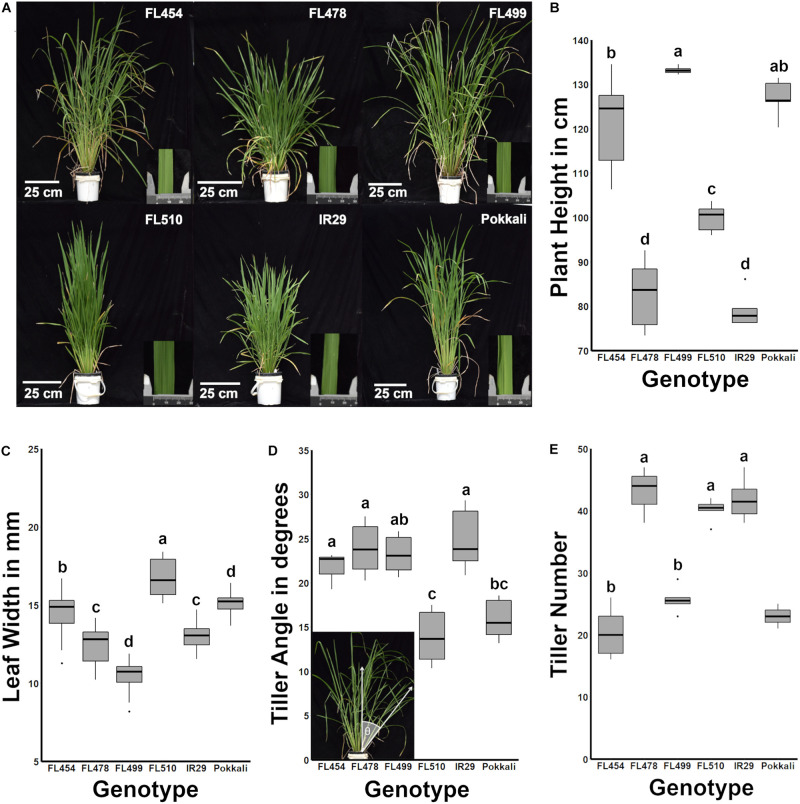
Morphological and growth habit profiles across the IR29 × Pokkali minimal comparative panel. The genotypic panel was grown under optimal conditions and compared morphologically and physiologically. **(A)** Gross-morphological differences highlighted the unique architecture of FL510. The inset photograph beside each plant shows a representative leaf under a caliper (units in mm). **(B)** Similarities across genotypes based on plant height (*P* < 0.05). FL454 and FL499, which are both sensitive to salt, are more similar to the tolerant donor parent Pokkali in terms of plant height, while FL478 is more similar to IR29. In contrast, FL510 is uniquely intermediate between the parents. **(C)** Box plots of leaf blade widths (*n* = 18) highlighting the uniqueness of FL510. In comparison, FL454 and FL478 are similar to the parents Pokkali and IR29, respectively. The super-sensitive FL499 has narrower leaves. **(D)** The tiller angle of each genotype (*n* = 6) was measured as the angle of the farthest tiller to its base as demonstrated in the inset photo and are shown as box plots. FL454, FL478, and FL499 are not significantly different from IR29 and have wider tiller angles compared to Pokkali and FL510 (*P* < 0.05). Tiller angle in FL499 was similar to Pokkali although it has a much higher mean value. **(E)** Number of tillers per plant showed that the genotypes with smaller statures (IR29, FL478, FL510) are more similar to each other with significantly higher values than the large-stature genotypes (Pokkali, FL454, FL499; *P* < 0.05). Taken together, morphological profiles indicate a compact architecture of FL510.

A key observation was the unique developmental and morphological attributes that highlight the transgressive nature of FL510. In terms of overall morphology and growth habit, the representative RILs in the panel were either similar to one of the parents (FL478 is similar to IR29) or hybrid for parental attributes (FL454, FL499). The clear exception was FL510, which had a modified architecture that resembled neither of the parents (Figure 1A). FL478 and IR29 had semi-dwarf stature (average height of 82.72 and 78.94 cm, respectively) with profuse tillering habit, characteristics of the Green Revolution plant architecture (Figure 1B). FL454 and FL499 had tall statures (average height of 121.17 and 133.20 cm, respectively) with slender and lanky leaves similar to Pokkali, and intermediate tillering habit. Distinct developmental and morphological features of FL510 were highlighted by intermediate height (average of 99.88 cm), low tiller angle, and thick, erect, dark green leaves, similar to the NPT.

The leaf morphology of FL454 based on width of leaf blades (average of 14.54 mm) appeared to have been inherited from Pokkali, while the FL478 leaf morphology resembled that of IR29. FL510 and FL499 were clear transgressives for leaf morphology having significantly wider blades (average width of 16.69 mm) than Pokkali, and narrower (average width of 10.46 mm) than IR29, respectively (Figure 1C). Width of leaf blades is known to contribute to higher transpiration rates in maple (Bauerle and Bowden, 2011). In rice, it has been associated with better yield, suggesting its positive contributions to photosynthetic capacity (Fujita et al., 2009). Wider leaf blade is also inversely correlated with leaf rolling under drought, suggesting that wider leaves contribute to osmotic stress tolerance (Cal et al., 2019).

Aside from thick, dark green, erect leaves, the tiller angle measured as the angle from the main axis of the plant perpendicular to the ground profoundly differentiated the transgressive super-tolerant FL510 from its parents and siblings. While FL454, FL478, and FL499 had similar angles as IR29, by virtue of its erect growth habit, FL510 had the smallest tiller angle even in comparison to Pokkali, which had the smaller angle of the two parents (Figure 1D). The uniquely compact stature of FL510 was also supported by tiller size and number, having the smallest mean tiller number among the smaller-stature genotypes. The other RILs with larger stature had lower tiller number, yet more spreading growth habit. IR29 and FL478 had smaller tillers with wider growth angle, which created a more open architecture and larger surface area (Figure 1E). Based on morphology and vegetative growth habit (tillering), the transgressive super-tolerant FL510 is unique by virtue of its compact, erect stature and intermediate height, all of which contribute to smaller surface area.

### Potential Effects of Plant Morphology on Water Retention

**FIGURE 2:**
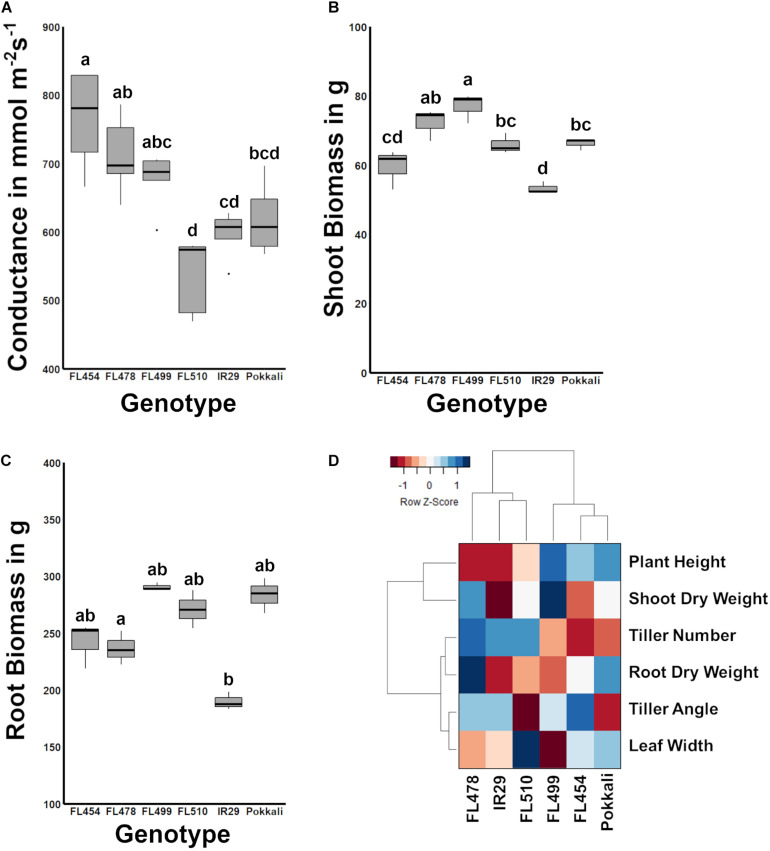
Comparison of stomatal conductance and biomass potential across the IR29 × Pokkali comparative panel, and measure of overall relatedness across the panel based on morpho-developmental and physiological profiles. **(A)** The width of leaf blades and tiller angle consequently affect transpiration. FL510, which had the smallest tiller angle and broadest leaf blades also had the smallest mean stomatal conductance, significantly lower than the other recombinant inbred lines (RILs; *P* < 0.05). While the stomatal conductance of FL510 was not statistically different from IR29 and Pokkali, it was lowest mean among the genotypes. **(B)** Shoot biomass indicated that FL510 had smaller exposed surface area compared to the other genotypes, while its shoot dry weight was comparable to the other genotypes. **(C)** Root biomass was not significantly different among the different genotypes, indicating that the difference in architecture is mainly due to shoot growth. **(D)** Hierarchical clustering dendrogram of morpho-physiological traits highlighting the similarity of FL510 and FL478 to IR29, and the similarity of FL454 and FL499 to Pokkali. The heatmap summarizes the widest deviation of FL510, which lie on its wider leaves and lesser tiller angle, to the common architecture of FL478 and IR29.

Leaf angle and how it shapes the overall surface area of the plant is an important aspect of transpiration. Upright (erect) leaf growth or tiller growth in the case of rice has been correlated with lower rates of transpiration than leaves or tillers growing at wider angles (Van Zanten et al., 2010). To further investigate the potential significance of the novel morphology and growth habit of FL510 to its unique ability to maintain robust growth under salt stress, the stomatal conductance was compared across the panel as a measure of transpiration rate. Results showed that FL510 indeed had the lowest stomatal conductance, significantly different from its sibling RILs (Figure 2A). The highest stomatal conductance was observed in the salt sensitive FL454, which also had among the widest tiller angles and narrowest leaf blades across the panel. The tolerant FL478 and super-sensitive FL499, both of which had tiller angle comparable to FL454, had the next highest stomatal conductance. These results are consistent with the inverse trends between morphology and stomatal conductance as observed in the other RILs.

In terms of biomass, FL499 had the highest shoot dry weight while IR29 had the smallest (Figure 2B). FL478 had slightly lower biomass than FL499 but was not statistically different from FL510 and Pokkali. FL454 was slightly smaller than the FL510 and Pokkali and was not statistically different from IR29. Differences in root biomass were not statistically significant (Figure 2C). The overall trends in shoot biomass are consistent with the uniquely compact architecture of FL510. Leaves growing at wider angles from the main axis of the plant have increased incidence of solar radiation, causing rapid elevation of internal leaf temperature and consequently, higher transpiration rates (Murchie et al., 1999; Van Zanten et al., 2010). The unique morphology and growth habit of the super-tolerant FL510, especially its narrow tiller angle appears to contribute to the reduction of the impact of osmotic stress under salinity as water is more efficiently retained by virtue of modulated transpiration. At the same time, the wider but erect, dark green leaves of FL510 appear to compensate for potential impediments to photosynthesis, despite reduction in gas intake. These features are likely allowing the plant to meet its energetic requirements while also modulating metabolic rate.

Traits that are most variable across genotypes (plant height, shoot dry weight, tiller angle, leaf width, and tiller number) were used to assess the overall developmental similarities across the panel. As shown in the hierarchical clustering dendrogram, the larger-stature (FL499, FL454, Pokkali) and smaller-stature (IR29, FL478, FL510) genotypes formed two distinct clades (Figure 2D). IR29 and FL478 were much closer to each other than FL510, due to the unique properties of FL510 for leaf width, tiller angle, plant height, and shoot dry weight. On the other hand, the main difference between Pokkali and the two larger-stature RILs was tiller angle, but with high similarity to FL454 with respect to other parameters. These results also highlight the uniqueness of the combination of traits that created FL510. While FL478 mainly inherited its morphology from IR29, FL510 combined the smaller stature of IR29 with a compact tiller angle of Pokkali. However, it also had transgressively wider leaves, which is likely a result of network rewiring.

### General Trends in the mRNA Transcriptomes of Recombinant Inbred Lines

Based on multiple layers of information from all available data (Figures 1, 2 in this paper and from Pabuayon et al., 2020), the overall morphology and growth habit of FL510 appeared to provide physiological advantage and flexibility under salt stress. This is likely through an inherent capacity to reduce the impact of osmotic stress while also maintaining adequate level of photosynthesis, hence avoiding severe penalties to growth. In combination with other defense mechanisms conferred by *SalTol* (Na^+^ exclusion), the transgressive super-tolerant FL510 appeared to have both the necessary repair/avoidance and physiological maintenance attributes to sustain better growth under severe salt stress, more so than its tolerant donor parent Pokkali and tolerant sibling FL478. To begin unraveling the genetic mechanisms behind such potentially unique synergy of growth, morphology, and physiological defenses in FL510, we conducted an in-depth profiling of the salt stress response mRNA transcriptomes across the comparative panel. The aim was to identify some of the critical regulators and targets (core and peripheral) involved in the expression of the inherent phenotypic potential of FL510.

**FIGURE 3:**
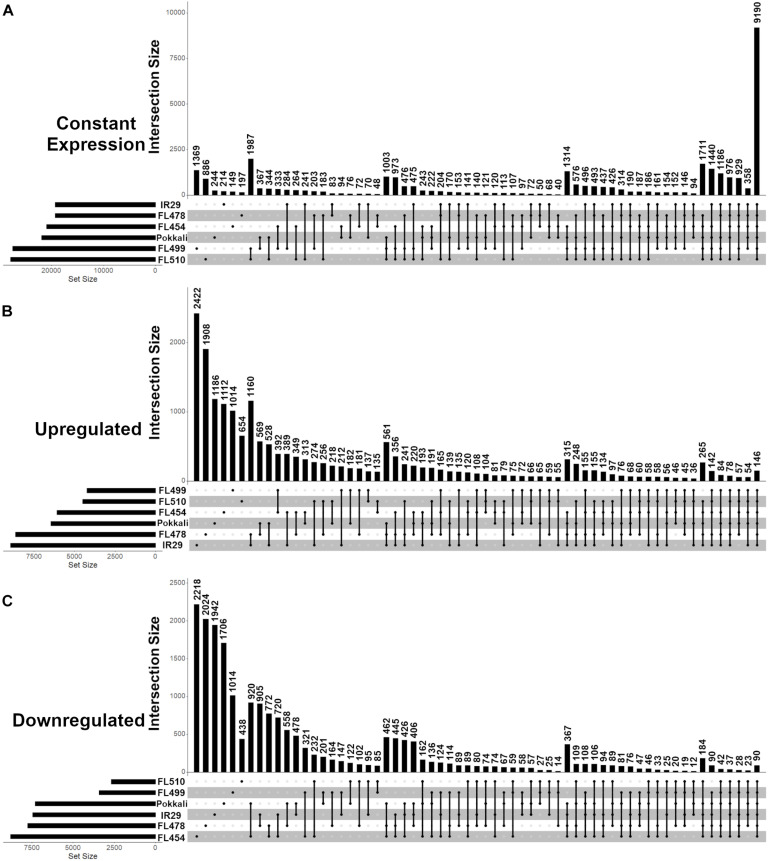
Distribution of protein-coding genes with unchanged (constant), upregulated or downregulated expression across the IR29 × Pokkali comparative panel as revealed by temporal RNA-Seq analysis before (0 h) and during (24, 48, 72, 144 h) salt stress at EC = 12 ds m^– 1^. Analysis was performed from V5 to V12 stage of vegetative growth. **(A)** Genes with unchanged (constant) expression had no significant changes in transcript abundance (< 2 and > -2 log2-fold) across all time-points. **(B)** Upregulated genes had significant increase in transcript abundance (> 2 log2-fold) relative to 0 h in at least one time point at EC = 12 ds m^– 1^. **(C)** Downregulated genes had significant decrease in transcript abundance (< -2 log2-fold) relative to 0 h in at least one time point at EC = 12 ds m^– 1^. Upset graphs accommodate all possible pair-wise comparisons and corresponding set intersections across pair-wise comparisons. The horizontal bar graph at the bottom left represents the total set size in each genotype, while the vertical bar graph represents the abundance of genes in each set, as dictated by the matrix at the bottom. The matrix indicates which genotype(s) combine for the vertical bar graph. The super-tolerant FL510 and super-sensitive FL499 had the least number of differentially expressed genes as shown in **(A–C)**. The tolerant FL478 and sensitive parent IR29 had the highest number of upregulated genes, followed by the tolerant donor parent Pokkali and sensitive FL454. Meanwhile, FL454 has the highest number of downregulated genes, followed by FL478, IR29, and Pokkali. The overlap between the subsets of stress responsive genes in FL510 and FL499 are minimal, indicating their responses are unique from each other.

The mapping statistics and coverage of the temporal mRNA-Seq datasets across the comparative panel before (0 h; control) and during (24, 48, 72, 144 h) salt stress (EC = 12 dS m^–1^) are summarized in Supplementary Table 1 (PRJNA378253: SRR11528266 to SRR11528295). Based on the distribution of mRNA abundances, the super-tolerant FL510 and super-sensitive FL499 appeared to exhibit the least changes in the transcriptome as indicated by the proportions of differentially expressed protein-coding genes (Figure 3A). The number of upregulated genes was highest in the tolerant FL478 and sensitive parent IR29 (Figure 3B), while the number of downregulated genes was highest in the sensitive FL454 (Figure 3C).

Common responses to EC = 12 dS m^–1^ were also evident across the comparative panel based on the overlapping patterns in gene expression. For example, the tolerant FL478 and its sensitive parent IR29 shared a total of 1,160 upregulated genes, which is more than double the number of upregulated genes shared by FL478 with its tolerant donor parent Pokkali (Figure 3B). Pokkali and IR29 had the third highest numbers of shared upregulated genes across all pair-wise comparisons, indicating major overlaps between their response mechanisms at least at the transcriptome level, despite their contrasting sensitivity to salt stress. In contrast, the transgressive super-tolerant FL510 was unique based on the small proportion of gene expression changes shared with any of the other genotypes. Consistent with its superior growth under EC = 12 dS m^–1^, it has the least overlap in gene expression with the sensitive FL454 and super-sensitive FL499. The two inferior RILs, i.e., sensitive FL454 and super-sensitive FL499, also shared the largest number of upregulated genes. Meanwhile, the tolerant donor parent Pokkali and its sensitive RIL FL454 had the largest number of shared downregulated genes, followed by IR29 and FL478 (Figure 3C). Pokkali and its sensitive RIL FL454 also exhibited similar proportions of downregulated genes, suggesting that downregulation of gene expression could be a critical component of the response mechanisms in Pokkali and FL454, more so than the other genotypes.

The transgressive super-tolerant FL510 and super-sensitive FL499 had the largest overlaps in their mRNA transcriptomes when the subset of genes whose expression did not changed at EC = 12 dS m^–1^ were considered (Figure 3A). The number of such genes is disproportionately large compared to any pair-wise comparison, consistent with the fact that the total number of differentially expressed genes were smallest in the two transgressive segregants. The implications of this common signature may be totally unrelated in FL510 and FL499, given their widely contrasting phenotypes at EC = 12 dS m^–1^. The small proportion of differentially expressed genes in FL510 may be an indication of less systemic perturbations, while the regulatory machinery for stress response may be inherently defective or inadequate in FL499.

**FIGURE 4:**
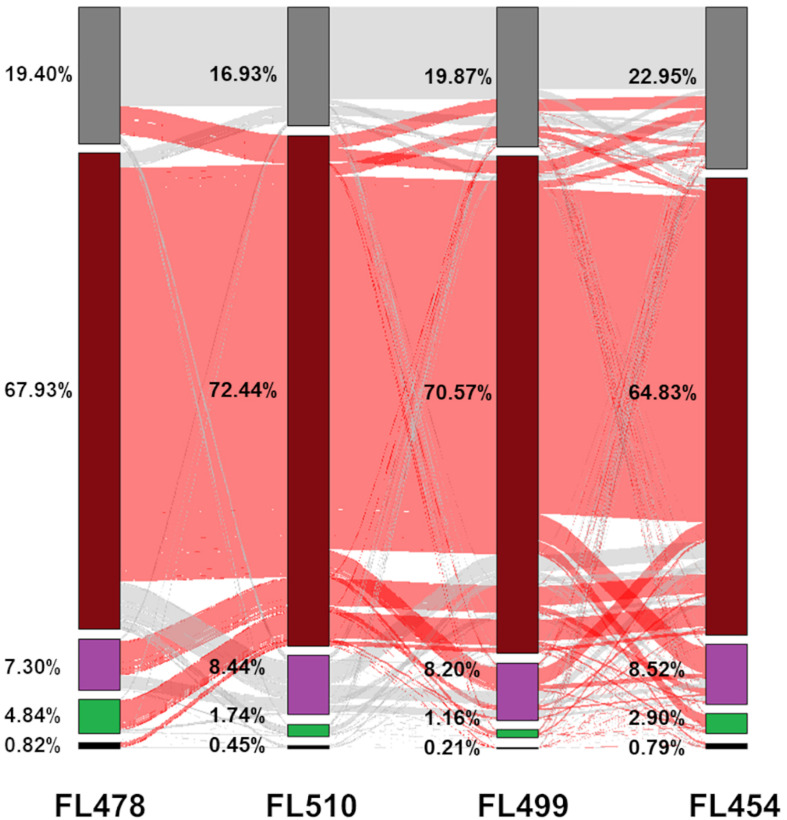
Analysis of the transmission of gene expression signatures from parents to offspring. Each protein-coding gene in the comparative transcriptome matrix was examined to trace the parental origin of respective expression patterns and classified into five categories: (1) complete inheritance (black), meaning that expression profiles were the same in both parents and in the RIL; (2) inherited from IR29 (green), meaning that the RIL had the same expression as IR29; (3) inherited from Pokkali (violet), meaning that the RIL had the same expression as Pokkali; (4) non-parental (brick red), meaning the expression pattern of the gene followed neither parent (deviant or transgressive); or (5) genes was excluded in the analysis due to very low transcript abundance (near zero) or extremely outlying values (gray). The proportion of genes shared with another category is denoted by the red and gray lines in between the bars.

Global transcriptome patterns (*n* = 34,935 unique protein-coding transcripts) were examined across the panel to trace the parental origins of the expression signatures in each RIL. FL510 and FL499 had the smallest numbers of genes that were not expressed in both control and stress conditions, compared to the other genotypes. General trends also highlighted the uniqueness of the two transgressive segregants by virtue of non-parental expression signatures in a large number of genes (Figure 4). For instance, the super-tolerant FL510 and super-sensitive FL499 had the smallest proportions of genes with the expected parental profiles, hence they also had the highest numbers of genes that deviated significantly from the expected parental profiles (transgressive). However, distinct subsets of genes were transgressively expressed in the super-tolerant FL510 and super-sensitive FL499.

The tolerant FL478 had the largest number of genes whose expression profiles were similar from either parent, of which a much greater number were from the sensitive parent IR29. This trend was not surprising given that FL478 is a specific selection in IRRI’s breeding program for the growth and developmental attributes of IR29, and limited introgression from Pokkali including *SalTol* (Walia et al., 2005; Cotsaftis et al., 2011; Chowdhury et al., 2016). Consistent with these trends, FL478 also had the smallest number of genes with Pokkali-type expression profiles (Figure 4). Meanwhile, the sensitive FL454 had the smallest number of genes with non-parental expression profile, and the highest number of genes with Pokkali-type expression profiles (Figure 4).

The patterns revealed by tracing the parental origins of expression signature for all annotated protein-coding genes in the transcriptome matrix are consistent with the true recombinant nature and genetic uniqueness of each RIL from the IR29 × Pokkali F_8_ population. In addition to expression signatures that were clearly inherited from either parent, each RIL showed significant deviations from parental gene expression profiles and the number of such deviant genes was highest among the two transgressive segregants at the opposite ends of the phenotypic spectrum. Trends uncovered from the global mRNA transcriptome data suggest that after eight rounds of recombination, network rewiring appeared to be most pronounced among the two phenotypic outliers in the population, i.e., FL510 and FL499.

### Cytokinin-Mediated Network May Contribute to Enhanced Growth Potential Under Salt Stress

The unique configuration of the regulatory network mediated by the Myb-type Multi-pass transcription factor *OsMPS* (Os02g0618400) in the transgressive super-tolerant FL510 has been previously reported (Schmidt et al., 2013; Pabuayon et al., 2020). It was proposed that the unique configuration of the *OsMPS* network in FL510 and its activation during the critical initial 24 h of salt stress facilitate an efficient integration of normal growth responses with stress responses, thus contributing to a large positive net gain in the overall potential of FL510. The unique molecular signature of FL510 as revealed by the global trends in the mRNA transcriptome (Figure 3) further supported the initial hypotheses of network rewiring (Pabuayon et al., 2020). To address this hypothesis further, we performed an integrative analysis of unique transcriptome signatures as a means to reveal the components of rewired networks associated with the novel growth and morphological attributes of FL510 (Figures 1, 2).

**FIGURE 5:**
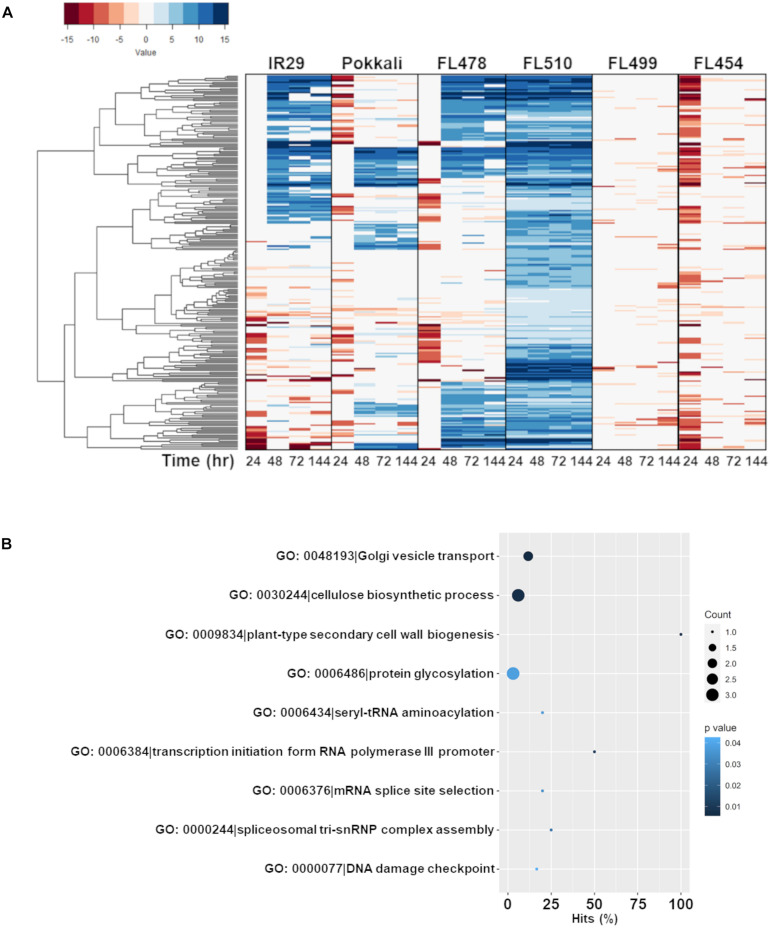
Comparison of the expression profiles of the subset of FL510-upregulated genes across all genotypes and their gene ontology enrichment. Genes that were upregulated by at least twofold (> 2 log2-FC) in FL510 across all time-points are included in this subset (*n* = 244). **(A)** Expression patterns of the subset of 244 genes across all six genotypes in IR29 × Pokkali RIL the comparative panel. While these genes had steady upregulation in FL510, only a fraction were upregulated in the other genotypes. Most of these genes were downregulated in super-sensitive FL499 and sensitive FL454. Upregulation of these genes were significantly delayed in sensitive parent IR29, tolerant donor parent Pokkali, and tolerant FL478. *Note: expression under control (0 h) is not displayed as expression data is shown as log2-fold expression relative to control.* **(B)** This cluster of FL510-upregulated genes is enriched with functions related to growth and cellulose deposition, indicative of continuous capacity for growth even under salt stress (*P* < 0.05).

Our approach was to mine the global transcriptome matrix across the comparative panel for co-expression modules that are unique to FL510 by PCC. The first level of selection for network components included genes that were upregulated by at least twofold (>2 log2-FC) across all time-points in FL510 in order to establish the total inducible transcriptome across all genotypes (Figure 5A). To establish the unique signatures of FL510, genes that made it to the initial shortlist was further filtered for those that were upregulated during the first 24 h of stress only in FL510 but downregulated in the inferior RILs. This excluded all other upregulated genes with potentially negative effects in the inferior RILs.

The first co-expressed cluster with a unique and transgressive signature in FL510 is comprise of 244 genes enriched with molecular and biological functions associated with the regulation of growth and development, and plant architecture (Figure 5B). The transgressive nature of this network in the super-tolerant FL510 was reiterated by the fact that FL478 was a clear combination of network signatures from both parents. This network of 244 genes appeared to be regulated primarily through the cytokinin signaling pathway as indicated by signature signaling genes such as *OsRR23* (Os02t0796500-01; B-type response regulator) and *OsHK5* (Os10t0362300-02; cytokinin signal receptor protein), biosynthetic genes such as *OsIPT9* (Os01t0968700-02; Isopentenyl transferase), and deactivation genes such as N-glucosyltransferase (Os03t0824600-00; cytokinin conjugation enzyme; Supplementary Table 2). The *OsIPT9, OsRR23*, and *OsHK5* have been reported to modulate adaptive morphology and growth under saline conditions, chemical toxicity, nutrient deficiency, and many biotic factors (Sharan et al., 2017; Ghosh et al., 2018; Nongpiur et al., 2019). The timely and robust expression of this cytokinin-mediated network in the transgressive super-tolerant FL510 is consistent with its unique adaptive morphology that may confer osmotic stress avoidance through regulated transpiration and robust photosynthesis (Figure 2).

Previous studies presented evidence that disjointed or fragmented organization of regulatory networks for growth adjustment and defense-related cellular process contributes to stress sensitivity in certain genotypes of rice (Kitazumi et al., 2018). To understand if the organization of the cytokinin-mediated network is a critical factor in the superior phenotype of FL510 and inferior phenotype of FL499 and other siblings, homologous network models were constructed across the comparative panel by PCC. Co-expression of orthologous genes was defined by network edges with high correlation coefficients of *r* ≤ -0.95 (inverse correlation) or *r* ≥ 0.95 (direct correlation).

**FIGURE 6:**
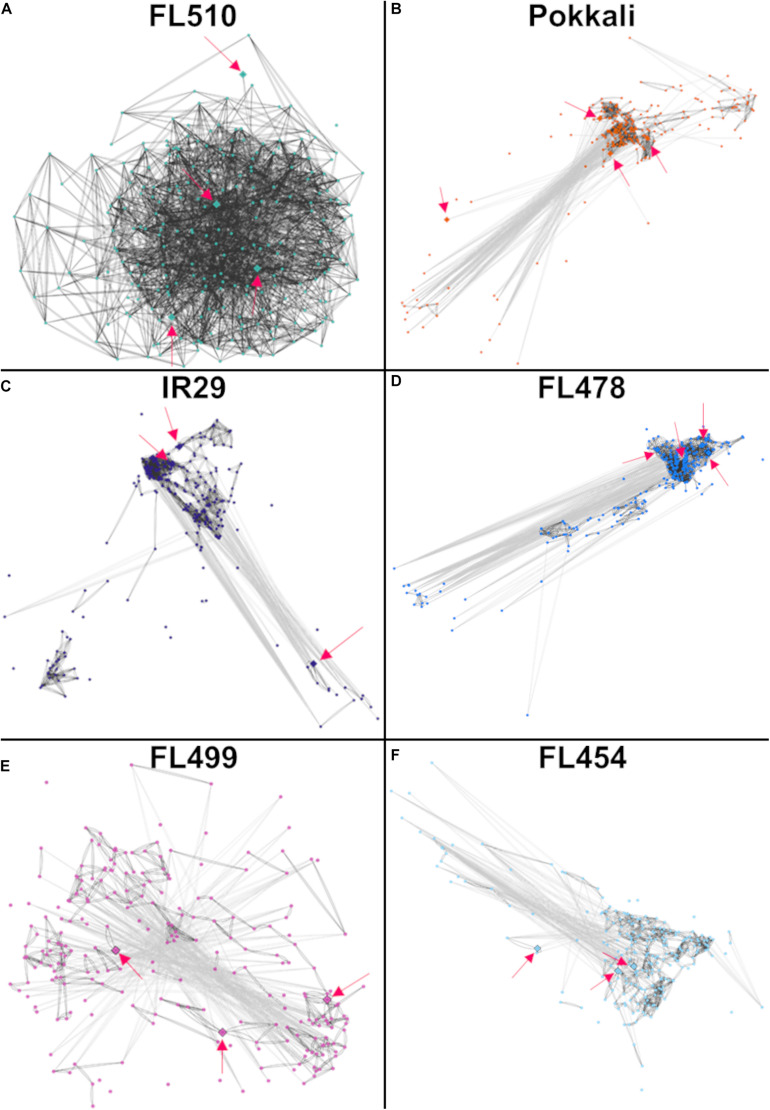
Models of the putative cytokinin-mediated transcriptional network comprised of 244 genes that were upregulated in FL510 as shown in Figure 5. Co-expression networks were constructed by Pearson correlation coefficients (PCC). Edges represent *r* values of at least -0.95 or 0.95. Dark gray lines indicate direct co-expression between two genes (nodes) and light gray lines indicate reverse co-expression. The distances between nodes are also reflective of the relationship between genes, with lower co-expression values having larger distances. Pink arrows indicate the position of the identified cytokinin-related genes, which are also denoted as diamond nodes. **(A)** The network in FL510 does not show negative co-expression between nodes. In contrast, the network appeared disjointed in **(B)** sensitive parent IR29, **(C)** tolerant donor parent Pokkali, and **(D)** tolerant FL478 with only partial conservation of the network connectivity observed in FL510. **(E)** The network in the super-sensitive FL499 is extensively fragmented, indicating a lack of functional coordination. **(F)** The network in the sensitive FL454 shows a different coordinated cluster that formed from their coordinated downregulation at 24 h. The inferior genotypes excluded one cytokinin-related genes (different in each genotype) as potential hub, further illustrating the fragmented nature of such networks relative to FL510.

The network model in the super-tolerant FL510 appeared to be well-connected based on consistently high *r* values, indicative of tight co-expression of component genes amongst each other and with their putative network hubs, *OsIPT9, OsRR23*, and *OsHK5* (Figure 6A). In contrast, the orthologous networks in other RILs and in the parents appeared fragmented and disorganized hence inferior to FL510 network (Figures 6B–F). Component genes in these networks form several smaller sub-clusters with different patterns of co-expression that deviate from the general patterns evident in FL510. In FL478, there are clusters missing from the main network relative to the network in FL510 (Figures 6B,D). Furthermore, some of network hubs appeared to be missing in the inferior genotypes (Figures 6C,E,F). Apparently, the deconstruction of the cytokinin-mediated network is worst in the inferior RILs. For instance, in the sensitive FL454, the component genes are not as tightly bound together compared with FL510 and FL478. There are many ‘*outlier or straggler*’ genes without significant linkages to the network because of weak *r* values. In the super-sensitive FL499, the network appeared to be lacking any order as most component genes had contrasting expression. This model suggests that the network is practically absent in this genotype. These results further illuminate why FL510 had minimal growth penalty, due to the optimal organization of growth-related genetic networks. Much of the growth enhancement of FL510 could also be attributed to its optimal architecture that provides better adaptive potential. Meanwhile, other genotypes, especially FL454 and the most inferior FL499, have high growth penalty due to the loss of synergy for an organized network.

Detailed examination of all functionally annotated genes in the FL510 network suggests an interplay of growth and development with mechanisms of stress mitigation by cross-talks with cytokinin signaling (Supplementary Table 2). For example, the regulatory transcription factor *OsHAP5J/OsNF-YC8* (Os01t0580400-01) plays an important role in both development and salt tolerance through ABA and other hormones (Alam et al., 2015; Li et al., 2016). The *OsWRKY13* (Os01t0750100-03) and *OsTGAP1/OsbZIP37* (Os04t0637000-02) transcription factors and the biosynthetic gene *OsOPR8* (Os02t0559400-01) are components of jasmonic acid (JA) response mechanisms, further reinforcing the importance of JA in the integration of growth and stress-related responses (Qiu et al., 2007; Yoshida et al., 2017). Network compositions also seem to suggest a potential interplay with brassinosteroid signaling, by virtue of the upregulation of *OsBZR4* (Os02t0233200-00; Bai et al., 2007).

### Genetic Network for Novel Adaptive Plant Architecture

**FIGURE 7:**
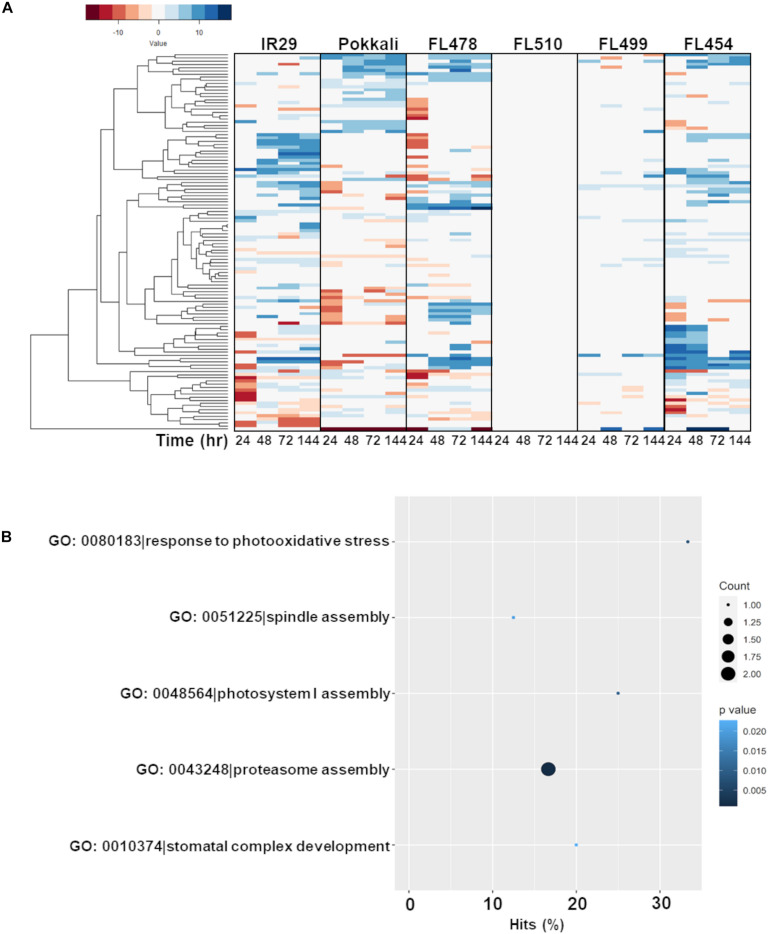
Comparison of expression profiles across all genotypes of the gene subset with constitutive expression only in FL510 and their gene ontology enrichment. Genes with consistent expression in FL510 across all time-points are included in this subset (*n* = 118). **(A)** Genes that had high expression in comparison to the parents under control conditions (>log2-FC at 0 h) and had small/no change in expression at different time points were selected for this analysis. Genes in this subset also showed differential expression in the other genotypes. Except for the super-sensitive FL499, other genotypes show different sub-clusters of co-expressed genes. *Note: expression under control (0 h) is not displayed as expression data is shown as log2-fold expression relative to control.* **(B)** Gene ontology analysis (*P* < 0.05) showed that this subset is enriched with molecular functions involved in growth and plant morphology, such as spindle assembly, photosystem I assembly, and stomatal complex development. This cluster also includes functions related to photo-oxidative stress response and proteasomal assembly.

The second cluster that formed a unique co-expression network in the transgressive super-tolerant FL510 is comprised of 118 genes with constitutively high expression (i.e., consistent expression not affected by salt stress) only in FL510 at a threshold of log2-FC > 2 (Figure 7A). We hypothesized that constitutive networks for the maintenance of plant form and growth habit might be critical to the unique adaptive morphology of FL510 (Figures 1, 2). Similar observations have been reported in *Solanum* and other angiosperms (De Almeida et al., 2014; Ichihashi et al., 2014). This network of 118 constitutively expressed genes is enriched with molecular functions such as response to photo-oxidative stress, proteasome assembly, spindle assembly, photosystem I assembly, and stomatal complex development (Figure 7B). Genes involved in floral organ development such as *FRIGIDA-*like protein (Os03t0794900-03), *HD3A* (Os06t0157700-01; heading date), and *OsFD5* (Os06t0724000-01; homolog of *OsFD1*) were also quite prominent in this network (Supplementary Table 3; Tamaki et al., 2007; Choi et al., 2011; Brambilla et al., 2017). These flowering regulator genes had stable expression in the transgressive super-tolerant FL510, but various patterns of upregulation were evident in the other genotypes, suggestive of perturbed developmental programming due to salt stress.

Most interestingly, enrichment of genes involved in the regulation of plant vegetative morphogenesis was quite evident in this network but only in FL510 (Figure 7A; Supplementary Table 3). This group includes prominent regulators of vegetative growth in grasses such as the transcription factor *OsIBH1* (Os04t0660100-02). The *OsIBH1* is a negative regulator of cell elongation through the brassinosteroid signaling pathway, leading to reduced tiller angle and erect growth habit. The effect of this transcription factor is modulated by negative regulators such as *OsBZR1* (Os07g0580500; *BRASSINAZOLE RESISTANT-1*) and *OsILI1/OsbHLH154* (Os04g0641700; *INCREASED LEAF INCLINATION 1*; Zhang et al., 2009). Also included in this cluster is another transcription factor *TAC3* (Os03t0726700-01; *TILLER ANGLE CONTROL-3*), which determines tiller angle in rice (Dong et al., 2016). The transgressive expression of these transcription factors in FL510, acting as potential hubs of vegetative morphogenesis network, are likely contributing to the unique morphology of FL510, which resembles the NPT architecture by virtue of its erect and broad leaves (Peng et al., 1994).

**FIGURE 8:**
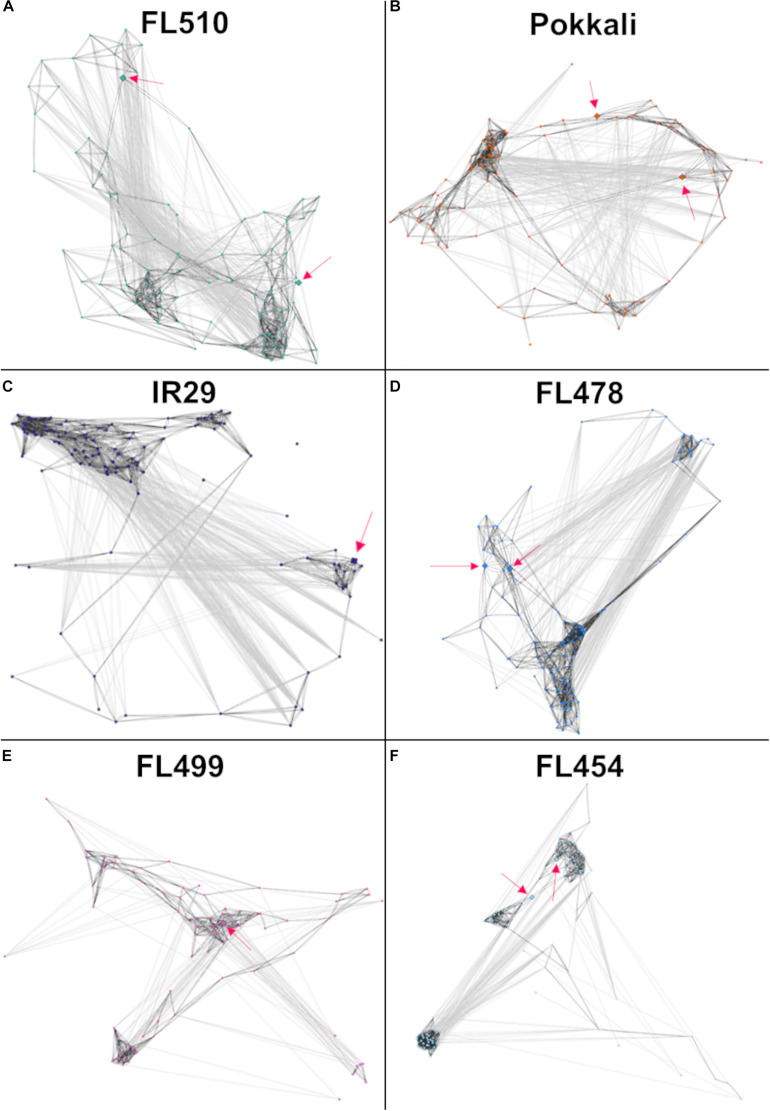
Models of the putative brassinosteroid-mediated transcriptional network comprised of 118 genes with constitutive expression only in FL510 as shown in Figure 7. Thresholds for PCC values were adjusted to -0.8 and 0.8, as this gene set had lower correlations due to constitutive expression in FL510. Dark gray lines indicate direct correlation, while light gray lines indicate inverse correlation. Pink arrows indicate the position of the two key genes in this group, namely *OsIBH1* (Os04t0660100-02) and *TAC3* (Os03t0726700-01), which are connected to brassinosteroid signaling and likely network hubs. **(A)** The network of FL510 is shown as a reference well-organized network in comparisons with the other genotypes. Various magnitudes of network fragmentation are shown for **(B)** sensitive parent IR29, **(C)** tolerant donor parent Pokkali, **(D)** tolerant FL478, **(E)** super-sensitive FL499, and **(F)** sensitive FL454. In all the genotypes, strongly co-expressed clusters can be observed. While still showing negative correlations, all the genes are still connected through positive co-expression in FL510 and Pokkali. In the other genotypes, the connectivity is significantly reduced or absent, creating clusters that have opposite expression patterns. These networks may indicate that these gene clusters are not unique among the different genotypes, but rather it is their constant expression in FL510 that makes it distinct.

Models of the constitutive vegetative morphogenesis network potentially mediated by *OsIBH1* and *TAC3* transcription factors through the brassinosteroid signaling pathway were constructed across the comparative panel in order to understand its potential significance to the unique attributes of FL510 (Figure 8). A modified threshold for constitutively expressed genes of *r* ≤ − 0.8 (inverse correlation) or *r* ≥ 0.8 (direct correlation) was used for this analysis. In FL510, all the nodes are connected by edges indicating strong co-expression (dark gray lines; Figure 8A). Except for the tolerant donor parent Pokkali, the other genotypes displayed a lack of inter-connectivity among the smaller sub-clusters, due to the strong co-expression within each sub-cluster or reverse co-expression between the small sub-clusters (Figures 8B–F). This trend reflects the similar trends observed in the cytokinin-mediated network for growth regulation (Figure 6), depicting the lack of coordination among the network components in the inferior genotypes. These trends also suggest the unperturbed nature of vegetative growth and morphogenesis in FL510 compared to the other genotypes.

The small tiller angle and erect leaf growth in the super-tolerant FL510 could have a major contribution to its ability to mitigate the effects of Na^+^ toxicity, as an offshoot of decreased water content from osmotic stress (Munns and Tester, 2008). The ability of a plant to exclude Na^+^ from functional plant tissues may be supplemented by the ability to retain water to further reduce cellular Na^+^ concentration. This may occur through a reduced transpiration rate, which also limits the uptake of solutes including Na^+^(Figure 2A). Thus, osmotic stress may be reduced by the inherent advantages from the unique architecture of FL510. This may be aided by sustained expression of other genes involved in cuticle formation such as *OsHSD1/LGF1* (Os11t0499600-01; hydroxysteroid dehydrogenase; Supplementary Table 1; Zhang et al., 2016). Genes involved in cutin, suberin, and wax biosynthesis showed sustained expression in the super-tolerant FL510 and its tolerant sibling FL478 (Supplementary Figure 1). Taken together, the transgressive nature of the constitutive brassinosteroid vegetative morphogenesis network suggests that the novel architecture of FL510 may complement the defenses acquired from Pokkali to minimize metabolic requirements under marginal conditions.

### miRNA Signatures Associated With Transgressive Salt Tolerance

**FIGURE 9:**
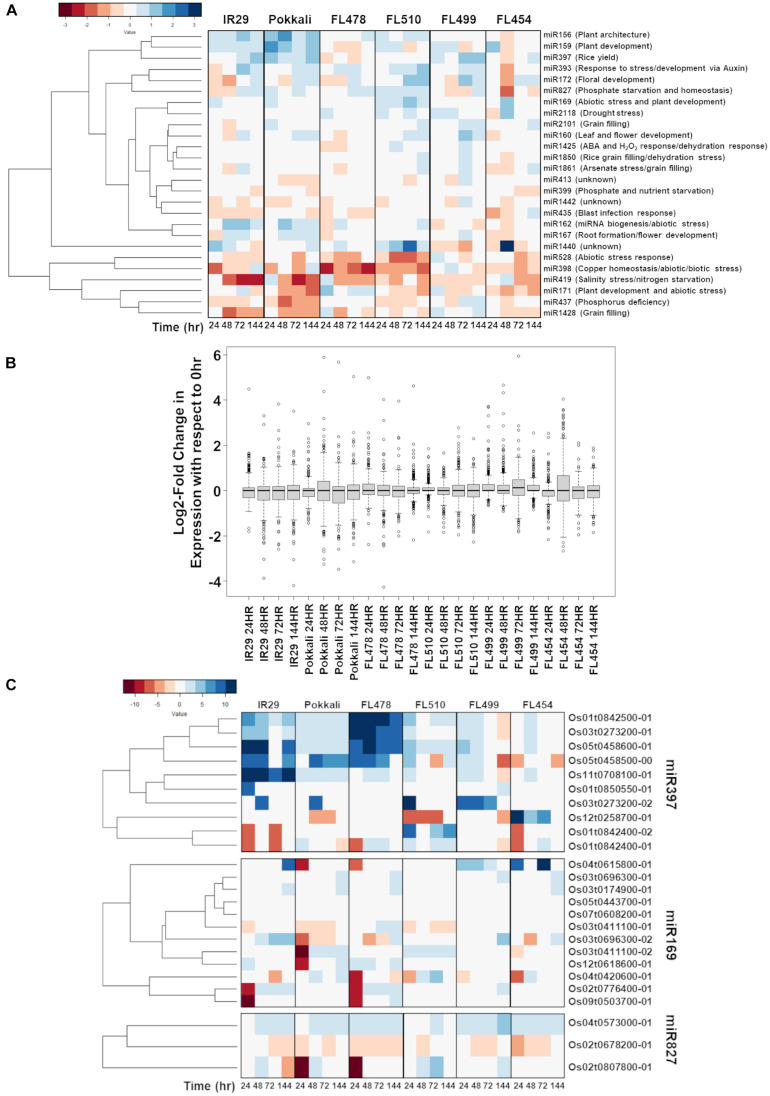
Comparison of the temporal co-expression of major miRNA families and their target genes across the comparative panel of IR29 × Pokkali recombinant inbred lines (RILs). **(A)** Mean expression of all miRNAs detected by RNA-Seq sequencing according to the classification of agronomically important miRNAs (Zhang et al., 2017) to minimize overlap between highly similar but distinctly annotated miRNA. **(B)** The miRNA expression across the different genotypes is similar, but expression in FL510 had smaller magnitude of changes with respect to control (0 h; i.e., narrow ranges). *miR169*, *miR827*, and *miR397* were uniquely expressed in the super-tolerant FL510. In comparison, the other genotypes have much wider ranges of values. **(C)** Expression of the downstream targets of miRNAs across the comparative panel. The *miR397*-target laccase genes were highly upregulated in FL478 and IR29, which suggest flavonoid oxidation as a response to stress. In FL510, the transgressive downregulation of *OsLAC29* (Os12t0258700-01) is consistent with its reported upregulation in salt-sensitive rice cultivars. *OsLAC4* (Os01t0842400-01, Os01t0842400-02) was transgressively upregulated in FL510, consistent with its reported function in maintaining structural integrity of leaves in rice. Differential expression of *OsHAP2E* (Os03t0411100-01, Os03t0411100-02) Pokkali and FL510 suggests targeted regulation of specific splice variant. *OsSPX-MFS1*,*2* (Os04t0573000-01, Os02t0678200-01) genes showed differential regulation except in FL510.

A series of miRNA-Seq libraries was also constructed in parallel to the mRNA-Seq libraries in order to reveal potential post-transcriptional regulatory signatures associated with transgressive phenotypes. The mapping statistics and coverage of the miRNA-Seq libraries are summarized in Supplementary Table 1 (PRJNA378253: SRR12213131 to SRR12213160). The list of non-redundant miRNAs expressed across the comparative matrix is summarized in Figure 9A according to the classification of agronomically important miRNA families (Zhang et al., 2017). While the great majority of salt stress-responsive miRNAs identified across the data matrix had strikingly similar expression profiles across the comparative panel, FL510 appeared to be the most unique by virtue of the narrow ranges of miRNA abundances compared (Figure 9B). This unique profile mirrors the more modulated changes in mRNA abundances in FL510 as shown in Figure 3, indicative of fine-tuned transcriptome and minimal system perturbation. In contrast, the other genotypes had wide ranges of miRNA abundances with extreme outliers, indicating sporadic changes in expression under salt stress (Figure 9B).

Among the shortlist of candidate salt stress-responsive miRNA families with unique signatures are *miR397*, *miR169*, and *miR827* (Zhang et al., 2017). *miR397* has a primarily developmental function and known as a positive regulator of grain size and panicle branching in grasses by modulating the expression of laccase genes (Zhang et al., 2013). Additionally, *miR397* has been reported to be involved in the inhibition of lignin production that supports secondary cell wall biogenesis and plant growth through cell expansion (Lu et al., 2013; Huang et al., 2016). Laccase genes are also involved in other types of defenses by acting on flavonoids (Turlapati et al., 2011). Upregulation of *miR397* at different temporal patterns was evident across the comparative panel except in the tolerant FL478 and super-tolerant FL510, where it was hardly detectable (Figure 9A). Given its possible role as a negative regulator of lignin production and secondary cell wall formation, the upregulation of *miR397* among the inferior RILs (FL454, FL499) with poor growth under salt stress, and its downregulation in the superior RILs (FL478, FL510) that grew better under salt stress, appeared to be correlated with the differential growth capacity of inferior and superior RILs.

A survey of the *miR397*-target laccase genes across the mRNA-Seq dataset showed strong upregulation particularly in IR29 and FL478 (Figure 9C). As the expression of laccase genes is strongly induced by salt stress in these genotypes, the cause may not be connected to lignin metabolism, but rather toward flavonoid oxidation processes as stress defense component. Meanwhile, Pokkali, FL499, and FL454 had strong upregulation of a laccase gene, i.e., *OsLAC14* (Os05t0458500-00) in Pokkali, *OsLAC11* (Os03t0273200-02) in FL499, and *OsLAC29* (Os12t0258700-01) in FL454. The *OsLAC29* was consistently downregulated in FL510, suggesting that this may be the main laccase gene affected by *miR397* induction. Upregulation of this laccase gene has also been reported in salt-sensitive rice cultivars, including IR29 (Liu et al., 2017). While the data presented does not show the same pattern observed in the previous study, upregulation in the sensitive FL454 and downregulation in the tolerant Pokkali and FL510 indicate that its expression may be detrimental toward tolerance. In contrast, *OsLAC4/OsLacc/OsLAC17* (Os01t0842400-01, Os01t0842400-02) showed transgressive upregulation in FL510. The *Arabidopsis* homolog of this gene is important for lignin deposition in stems to maintain normal growth (Berthet et al., 2011). In rice, this laccase gene was found to be downregulated in mutants with rolled leaf phenotype (Fang et al., 2012). This suggests that the unique expression of *OsLAC4* in FL510 may be relevant to its rigid architecture.

The *miR169* has been reported to improve drought tolerance when overexpressed in tomato by virtue of its roles in the regulation of osmotic imbalance caused by dehydration (Zhang et al., 2011). Sustained upregulation of *miR169* under salt stress was evident only in the super-tolerant FL510, reflecting a transgressive profile (Figure 9A). The unique upregulation of *miR169* in the super-tolerant FL510 may be suggestive of its possible contributions to osmotic adjustment mechanisms. Genes potentially targeted by *miR169* belong to the *Nuclear Factor Y (NF-Y)/Heme Activator Protein (HAP)* family. We found that the expressions of *miR169*-target genes were sparse especially in FL510, FL454, and FL499 (Figure 9C). However, the expression of *OsHAP2E* (Os03t0411100-01, Os03t0411100-02) in FL510 and Pokkali were unique. The longer transcript variant (Os03t0411100-01) was downregulated, while the shorter (Os03t0411100-02) was upregulated, indicating a possible difference in their sensitivity to miRNA-directed degradation. *OsHAP2E* overexpression has been implicated with salinity and drought tolerance (Alam et al., 2015).

Similar to *miR169*, *miR827* was detected at high abundance across the entire duration of salt stress (sustained; transgressive) only in FL510. The *miR827* plays a role in the regulation of responses to phosphate starvation especially under salinity and drought (Lin et al., 2010; Kant et al., 2011; Ferdous et al., 2017; Shukla et al., 2018). The targets of *miR827* showed a unique expression in FL510 (Figure 9C). There was minimal change in *OsSPX-MFS1* and *2* (Os04t0573000-01 and Os02t0678200-01, respectively), while *OsWAK20* (Os02t0807800-01) was only upregulated in FL510. *OsSPX-MFS* genes are involved in phosphate starvation during salinity (Wang et al., 2015; Wang et al., 2017). It has also been reported that *OsSPX-MFS* genes are expressed in opposite patterns during phosphate starvation, with *OsSPX-MFS1* being downregulated and *OsSPX-MFS2* being upregulated (Lin et al., 2010). Expression of these genes in the inferior genotypes indicates that there is a surplus of phosphate requiring transport to the tonoplast to preserve homeostasis. In contrast, minimal changes in FL510 are indicative of less systemic perturbation. The specific co-upregulation only in the super-tolerant FL510 of two miRNA families (*miR169, miR827*) with shared roles in osmotic stress mechanisms is consistent with the growth and morphological attributes of FL510 (Figures 1, 2), implying potential significance to osmotic stress tolerance. Overall, the unique miRNA profiles of FL510 revealed another layer of information that points to the importance of osmotic stress tolerance to its total phenotypic potential, by virtue of its novel morphology and growth habit.

## Discussion

Transgressive segregation is observed both in nature and in artificial populations created by plant breeding. However, such individuals exceeding the parental phenotypic range arise only in very small proportions of the population thus also referred to as genetic novelties (Rieseberg et al., 1999; Wagner and Lynch, 2010). Transgressive segregation in natural populations have been proposed as an important driver of adaptive speciation through the nucleation of new phylogenetic lineages with novel ecological niche. This theory has important implications to modern plant breeding paradigms as crop domestication and improvement by directed mating aim for similar outcomes as adaptive speciation, i.e., crops that maintain productivity under marginal environments (de Los Reyes, 2019).

In light of the current paradigms for developing novel adaptive phenotypes for use in marginal agriculture, modern plant breeding and biotechnological approaches must be guided with how evolutionary processes lead to optimization and fine-tuning to create the most adapted individuals. The modern reductionist approach to genetic manipulation considered mostly the inducible defense components of such a complex trait as adaptation, while neglecting the importance of inherent constitutive morpho-developmental attributes as important aspects of adaptive traits. Looking back at the success of the Green Revolution in the 1960’s, classical ideotype breeding created the most optimal plant architecture to maximize plant productivity potential under water- and nutrient-rich environments (Hedden, 2003). During the more recent times, a NPT was created in rice that further limits wastage of resources on extensive vegetative growth by only producing few but productive tillers. It was successful in out-yielding *indica* rice cultivars and even spurred the creation of “super” rice hybrids in China (Laza et al., 2003; Peng et al., 2008). However, for salinity, morphological modifications are often overlooked, as the primary concern in mitigating its effects is excluding Na^+^. Therefore, salinity tolerance is thought to hinge upon the differences in the capacity of Na^+^ transporter alleles to translocate toxic ions. The classic paradigm of ideotype breeding must be reintegrated in modern biotechnology and genomics-based crop improvement to create the next generation of crops with high adaptive capacities to marginal conditions, in a manner that is guided by evolutionary principles.

The importance of novel adaptive morphology and development in re-envisioning the current approaches to crop genetic manipulation can best be illustrated by evolutionary examples. The evolution of halophytic plants is a good illustration of how specialized tissues and organs work in synergy with inducible genetic defense mechanisms to provide the plant the capacity to thrive under saline environments. Different species rely on different mechanisms such as succulence, salt secretion, or ion compartmentalization at much higher capacities than glycophytes (Flowers and Colmer, 2008, 2015). There are many commonalities in the mechanisms used by plants for survival under marginal environments (de los Reyes et al., 2018). However, halophytes expand on these common mechanisms through unique morphological modifications (Breckle, 2002). For example, halophytes can expand their capacity to absorb salt by developing succulent leaves or stems. Others like *Atriplex centralasiatica* develop salt glands to secrete NaCl from leaves (Yuan et al., 2016). *Porteresia coarctata* (syn: *Oryza coarctata*) has the capacity to excrete salt from its leaves through microscopic hairs (Flowers et al., 1990; Brar et al., 1997).

Improving plant performance through morphological modifications has been done for a variety of conditions. For example, *OsSPL14* has been used to improve panicle branching and increase yields in rice (Miura et al., 2010). Morphological traits have been used in conjunction with molecular markers to improve drought tolerance (Manickavelu et al., 2006). More prominently, root system architecture has always been a popular target for improving yield performance under drought (Henry et al., 2011; Kulkarni et al., 2017; Ye et al., 2018). In some cases, characteristics that may impress as very minor can have profound effects in agronomic performance, as in the case of the *DRO1* gene for root architecture (Uga et al., 2013).

Results of this study point to the importance of plant architectural modification created through transgressive segregation, an important process in natural adaptive evolution, in complementing the effects of inducible defenses against salt stress. In the previous study conducted on the same genotypic comparative panel, it was hypothesized that FL510 was more metabolically efficient under salinity stress compared to its siblings and parents (Pabuayon et al., 2020). Both FL510 and FL478 inherited a superior Na^+^ exclusion mechanism from the donor parent Pokkali; however, the mechanism of Na^+^ exclusion will eventually become overloaded, unsustainable, and ineffective under long-term salinity stress. It will then become necessary to prolong the efficacy of Na^+^ exclusion to extend survival through other means that may be directly or indirectly connected to inducible defenses. By identifying such mechanisms, many of the contributing components behind the transgressive nature of FL510 have been illuminated.

**FIGURE 10:**
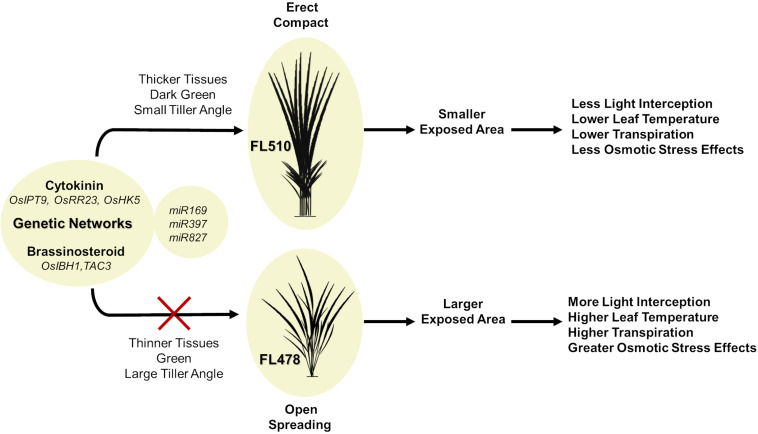
Hypothetical model summarizing the potential contributions of two intact genetic networks (i.e., putative cytokinin-mediated and brassionosteroid-mediated networks) to the maintenance of total adaptive potential of FL510 under salt stress through the integration of morpho-developmental with physiological defense attributes. The model shows the comparison of the outcomes of a novel compact plant architecture (FL510) with conventional dispersed or open growth habit (FL478). More compact structure in FL510 conferred by the putative cytokinin and brassinosteroid-mediated networks (Figure 8) leads to better defenses against osmotic stress. Smaller tiller angle lead to lower transpiration rate, and thus water loss is limited. This is an important advantage, as an open architecture (FL478) will lose water faster but will not be able to replenish it as it would increase Na^+^ uptake.

A hypothetical model that integrates the different components behind the novelty of transgressive segregants is shown in Figure 10. The most prominent feature that makes FL510 unique is its plant architecture. Its compact structure is the result of combining the smaller stature of its salt-sensitive parent IR29 and the low tiller angle property of its salt-tolerant parent Pokkali. Additionally, its height is intermediate, and its leaf width is transgressively larger compared to its parents and other siblings as a consequence of network rewiring. Studies on leaf width and its association with drought stress suggest critical roles in osmotic stress tolerance and lesser degree of leaf rolling (Cal et al., 2019). Compact structure confers an advantage through the lowering of transpiration rate compared to individuals with more open growth habit. While the open structure allows for more light interception, this also has a trade-off of being more exposed for gas exchange, which allows more water to be released to the air. Under saline conditions, this can be a disadvantage, as water loss translates to increased cellular Na^+^ concentration. Uptake for water (solute) will also inevitably bring in more Na^+^, thereby further necessitating Na^+^ exclusion, which also brings to a risk of exhausting such capacity faster. If water uptake is limited, the plant experiences osmotic stress, with immediate effects on growth capacity (Munns and Tester, 2008). In contrast, a compact plant architecture retains water more efficiently because of smaller exposed surface area that lowers leaf internal temperature and transpiration rate. This mitigates the effects osmotic stress, allowing a longer period by which water uptake and consequently Na^+^ intake could be limited. The net result is the prolongation of the effectiveness of Na^+^ exclusion mechanism. While these factors do not directly affect Na^+^ exclusion mechanism *per se*, they are necessary to preserve efficacy and help extend the time duration until perturbation and injuries could no longer be controlled.

We reported earlier that except for FL510 and FL499, most RILs derived from IR29 × Pokkali had intermittent changes in gene expression especially at the short-term (Pabuayon et al., 2020). In contrast, there was minimal induction or repression of genes under salinity in FL510. Genes that were transgressively upregulated in FL510 within the critical first 24 h of salt stress had mainly growth-related functions. This indicates that FL510 either did not experience the same magnitude of stress like the other genotypes, or it continued growth to enhance its capacity to sustain itself despite the sub-optimal conditions. New growth also allows accumulation of more Na^+^ in older leaves, assuring the continued function of younger and more active leaves (Yeo and Flowers, 1982). It is also important to have adequate supply of photosynthate in source organs to fuel reproductive growth and grain filling. Immediate shift toward creating new tissues and organs instead of entering stasis, as the case was in the inferior genotypes, may have prevented additional accumulation of Na^+^ that would hamper other cellular functions. While the parents and tolerant sibling FL478 showed upregulation of portions of the network in FL510 at much later time-points, such limited responses apparently did not elicit the same positive effects as in FL510. This study also points to the importance of maintaining constitutive networks that sustain morphogenesis and growth. Typically, genes which are responsive to stress are given more stock as it is often perceived that response equates to adaptation. However, genes that confer adaptation even before the onset of stress could be expressed steadily. Two genes that are potential culprits of the unique architecture of FL510, *OsIBH1* and *TAC3*, were in fact constitutively and transgressively expressed.

It is also important to underscore the additive nature of the genetic networks involved in plant morphology and growth under stress. Co-expression networks allow the visualization of the interconnectivity between genes that may or may not have direct effect with each other, consistent with the Omnigenic theory. It also forms a baseline in determining which genes potentially interact with one another that can be further supplemented with other gene interaction data. In this study, the key genes involved in the cytokinin-mediated network were detected as a complete set in the superior genotypes, but not in the inferior genotypes (Figure 6). Additionally, in the tolerant but non-transgressive FL478, the core genes had strong co-expression, but the network was incomplete due to gene sub-clusters that were inversely expressed relative to the major hubs. The same pattern was evident in the putative brassinosteroid-mediated network (Figure 8). In the inferior genotypes IR29 and FL499, one of either *TAC3* or *OsIBH1* transcription factors that may be functioning as hubs of the network was missing. Therefore, maximizing the genetic potential of a plant entails having intricately coordinated expression of many genes that form a network, as observed in FL510. These results point to the cumulative effects of different components the network, consistent with the *Omnigenic Theory*. It may also be possible to use the results from the network analysis to model and quantify the phenotypic effects of synchronized expression in a network, or as training model for genomic selection.

The miRNA genes that were transgressively expressed in the super-tolerant FL510 provide additional support to the ability to mitigate the initial impact of salinity through osmotic effects. Early and consistent upregulation of *miR169* and *miR827* in FL510 but not in the other genotypes suggest that the initial response was active rather than the passive (Pabuayon et al., 2020). This may also explain the low number of differentially expressed genes in FL510, as its response is finely regulated and targeted, requiring only a much smaller group of genes. Specifically, regulation of *OsLAC4* is consistent with the integration of growth and stress responses. The regulation of *OsSPX-MFS* genes also provides an insight into the robust nature of FL510. Phosphate homeostasis is important under salinity stress, as excess phosphate can inhibit growth, compounding the effects of increased salt concentration (Aslam et al., 1996). In comparison, there is minimal regulation of these genes in FL510 suggesting a relatively unperturbed phosphate homeostasis. Tolerance is often associated with survival rate under unfavorable conditions. However, in the case of FL510, tolerance translates more toward minimal system perturbations. Externally, this is manifested by low growth penalty, and internally by more refined changes in the transcriptome. System perturbations exacerbate the injuries as reactions can be energetically wasteful.

Fine-scale characterization of a transgressive segregant such as FL510 opens new paradigms in breeding for adaptive traits since novel phenotypes can arise through optimal complementation of different traits that may otherwise seem trivial. Repeated recombination events provide more genomic permutations that could lead to genetic novelties by network rewiring. Thus, outlier individuals arising from a population, while seemingly unimportant, can provide insights to different types of possible synergism that are optimal under different environments. This study also points to the importance of traits that are often overlooked. For example, during selection in plant breeding, the capacity to sustain growth under salinity stress is often viewed of secondary importance to Na^+^ exclusion capacity. However, the two traits should be complementary, as one trait feeds and expands the capacity of the other. This concept goes back to the *Omnigenic Theory*, which points to seemingly unimportant peripheral genes working in the background of the core genes to create a fine-tuned synergy (Boyle et al., 2017).

In this study, the fragmentation of gene networks in inferior genotypes is suggestive of a loss of contributory peripheral genes, with cumulative effects toward lowering the overall adaptive potential of the plant. Complete networks in FL510 is necessary for maximized phenotypic performance. Therefore, manipulation or addition of singular alleles or genes will not maximize a plant’s potential. It is through the synergy of the entire system that the overall phenotypic potential can be fully realized, and this is possible only through the process of genetic recombination. This study also highlights the power of genetic recombination to produce phenotypes which appear to be combinations of minute and indistinguishable traits from the parents yet creating to novel morphologies. The next step to ascertain the effects of these genes would be to investigate loss of function or gain of function mutants with an aim of reconstructing similar morpho-developmental features as FL510. While this is exciting at a theoretical level, it may be challenging to achieve given that FL510 has a unique stacking of beneficial traits. A more feasible approach for further validation would be the analysis of interactome networks, which would entail cis-element and protein-protein interaction analysis. Additionally, the unique trait configuration of FL510 means that the best way to validate the results from this study is to be able to select new genotypes using the genomic model of FL510.

The study also presents a new viewpoint in addressing the problem of what traits should be targeted for crop improvement. Since abiotic stress conditions are multifaceted, approaches for mitigation should also attempt to cover each effect as much as possible. Addressing only one part of the issue, such as using single major QTL is insufficient. Additionally, it is necessary to not just examine phenotypes under stress but also under normal states to discover new mechanisms that can be utilized to improve phenotypic performance. It is necessary to determine the traits that are complementary to the baseline mechanisms (de los Reyes et al., 2018). The study contributes to a comprehensive pipeline for creating models for larger populations in the future, for genomic selection of new genotypes with maximized phenotypic performance. Traits do not operate in a vacuum with respect to each other. Rather, they act in synergy with one another, or can act antagonistically.

## Data Availability Statement

The RNA-Seq data used in this article are publicly available in the NCBI Short Read Archive (PRJNA378253: SRR11528266–SRR11528295 and SRR12213131–SRR12213160).

## Author Contributions

IP, AK, and BR designed the experiments and wrote the manuscript. IP and AK performed the experiments and data analysis. RS and GG created the recombinant inbred populations and generated initial plant phenotyping data. BR conceptualized the project and designed the experiments. All authors contributed to the article and approved the submitted version.

## Conflict of Interest

The authors declare that the research was conducted in the absence of any commercial or financial relationships that could be construed as a potential conflict of interest.

## Supplementary Material

Supplementary Figure 1 Expression of genes related cutin, suberin, and wax biosynthesis across the different genotypes used in the study. Genes that belong to this biosynthetic pathway in KEGG (ko00073) were surveyed for their expression across the genotypes in the panel and were clustered together hierarchically. Among the genotypes, the two tolerant recombinant inbred lines (RILs) FL510 and FL478 mainly showed upregulation, while the others had both up- and downregulation.

Supplementary Table 1 Summary and mapping statistics of the RNA-seq libraries constructed, processed, and analyzed for the parental genotypes and selected recombinant inbred lines (RILs).

Supplementary Table 2 Genes uniquely upregulated in FL510 at all timepoints immediately after imposition of stress and downregulated in sensitive genotypes FL499 and FL454.

Supplementary Table 3 Genes that show high basal expression in FL510 compared to the parental lines (log2-FC > 2) and showed no differential expression under salinity stress.
