# Supplementary figures and images for: Contributions of Adaptive Plant Architecture to Transgressive Salinity Tolerance in Recombinant Inbred Lines of Rice: Molecular Mechanisms Based on Transcriptional Networks
Source: Front Genet. 2020 Oct 23;11:594569. doi: 10.3389/fgene.2020.594569 (PMC7644915; doi:10.3389/fgene.2020.594569)

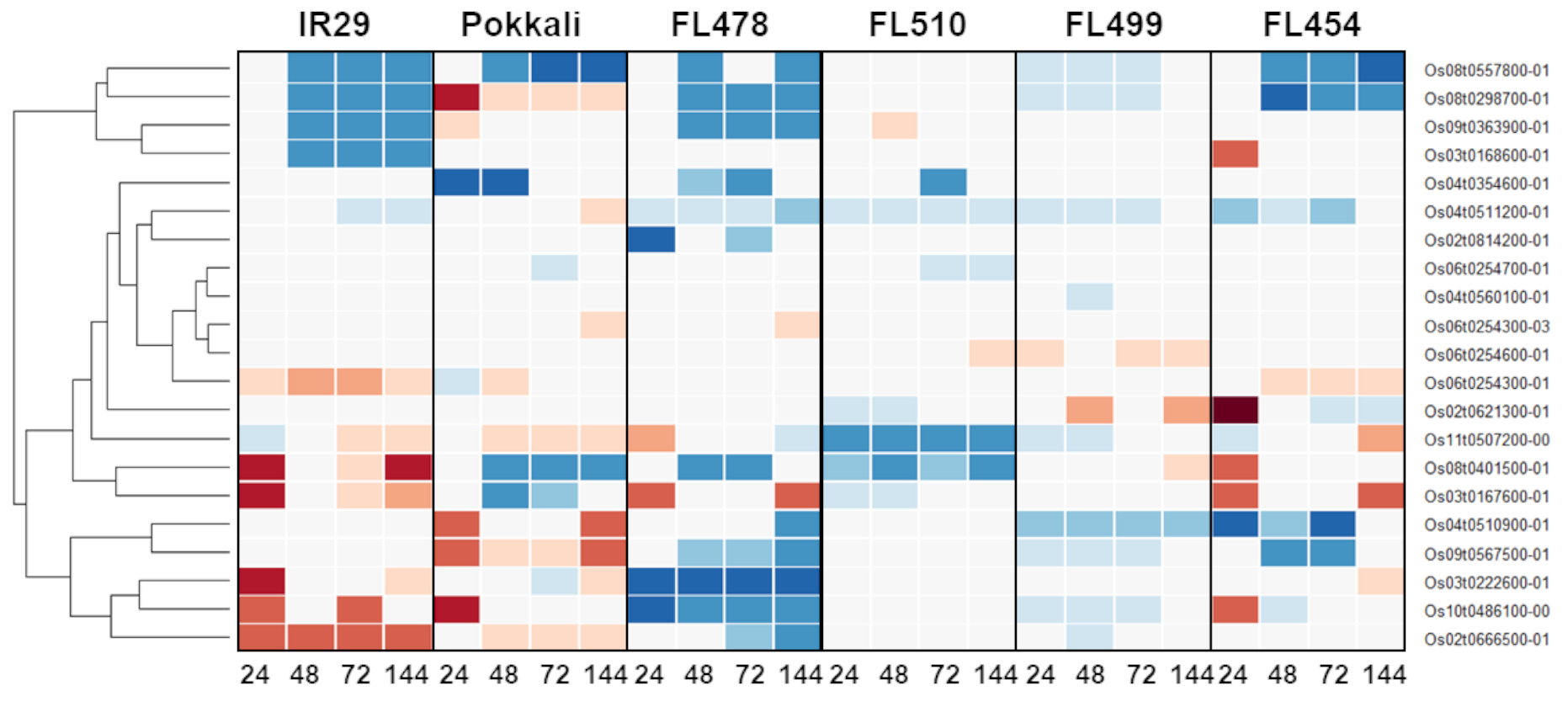

Supplement: Supplementary Figure 1 — Expression of genes related cutin, suberin, and wax biosynthesis across the different genotypes used in the study. Genes that belong to this biosynthetic pathway in KEGG (ko00073) were surveyed for their expression across the genotypes in the panel and were clustered together hierarchically. Among the genotypes, the two tolerant recombinant inbred lines (RILs) FL510 and FL478 mainly showed upregulation, while the others had both up- and downregulation. [file Image_1.JPEG]
